# Middle Ear Status in Cleft Lip and Palate Patients: A Five-Year Follow-Up 

**DOI:** 10.22038/IJORL.2022.62094.3134

**Published:** 2022-09

**Authors:** Fatemeh Mirashrafi, Hamed Emami, Zeinab Bagheri, Sara Rahavi-Ezabadi

**Affiliations:** 1 *Otorhinolaryngology Research Center, Otorhinolaryngology Head and Neck Surgery Department, Children’s Medical Center, Tehran University of Medical Sciences, Tehran, Iran.*; 2 *Otorhinolaryngology Research Center, Otorhinolaryngology Head and Neck Surgery Department, Imam Khomeini Hospital Complex, Tehran University of Medical Sciences, Tehran, Iran.*

**Keywords:** Middle ear status, Otitis media with effusion, Palatoplasty, Sommerlad technique, Ventilation tube

## Abstract

**Introduction:**

The best strategy to treat otitis media with effusion in cleft lip/palate patients is still under debate. This research aimed to evaluate the otologic outcomes in children at least five years post-repair.

**Materials and Methods:**

A retrospective study was conducted on 40 children who underwent palatoplasty between January 1, 2012, and January 1, 2014, at Children’s Medical Center (Tehran, Iran). Patients had intervelar veloplasty under magnification (Sommerlad’s Technique). Based on patients’ charts, their age, gender, cleft type, date of palatoplasty, as well as the date and the frequency of ventilation tube (VT) insertion, were recorded. Furthermore, otomicroscopy, middle ear status, and tympanometry were assessed five years postoperatively.

**Results:**

There was no significant difference in middle ear status between children with complete and incomplete cleft palates. The mean age at the time of study and the mean follow-up duration were significantly higher in the normal middle ear group, compared to the abnormal middle ear group (7.7±1.6 vs. 6.8±0.9, P=0.03 and 6±1.15 vs. 5.42±0.9, P=0.04, respectively). Middle ear status was not significantly different between early or late palatoplasty patients. In addition, the frequency and timing of VT insertion were not significantly different between the two groups.

**Conclusions:**

Middle ear status improved as patients grew older; however, the age of palatoplasty and the frequency of VT insertion were not significant prognostic factors in patients who underwent intervelar veloplasty under magnification.

## Introduction

Cleft lip/palate (CLP) is among the most frequent congenital disorders worldwide and is more prevalent among Asians and Native Americans (1). Various factors, such as alcohol consumption, smoking, steroids, viral infection, anticonvulsants, and retinoids, can cause CLP (2,3). Otitis media with effusion (OME) is a common condition among CLP patients that may affect speech, language, and learning development (4). Immature development of the Eustachian tube, abnormal formation of the tensor veli palatini muscle or levator veli palatini muscle, and craniofacial bone abnormalities can cause the development of recurrent or continuous OME in CLP patients. Previous studies have shown that 80% of CLP patients do not have OME at birth, but 90% of infants suffer from OME before their first birthday, and 97% experience OME within the first two years of their life (5-7). Recurrent or chronic OME can cause atelectasia, ossicular fixation, and/or tympanosclerosis. These pathologic changes cause conductive and sensorineural hearing loss at critical ages of development which may affect speech and language skills (8). Different factors, such as age at palatoplasty, the frequency of ventilation tube (VT) insertion, the type of cleft, surgical technique, and ethnicity, were key prognostic factors influencing hearing outcomes in CLP patients (9,10). This study aimed to evaluate the otologic outcomes in children at least five years post-repair. 

## Materials and Methods

A retrospective study was conducted on 40 children who underwent palatoplasty between January 1, 2012, and January 1, 2014, at Children’s Medical Center (Tehran, Iran). The study was approved by the Tehran University of Medical Sciences (IR.TUMS. MEDICINE. REC.1397.302). Patients had intervelar veloplasty under magnification (Sommerlad’s Technique). When necessary, lateral releasing incisions (Von Langenbeck flaps) were used to help close the entire palate. Patients with an identified sequence or syndrome were excluded from the study. Based on patients’ charts, their age, sex, cleft type, date of palatoplasty, as well as the date and the frequency of VT insertion, were recorded. Those with incomplete charts were also excluded from further analysis. Otomicroscopy, middle ear status, and tympanometry were assessed five years postoperatively. Furthermore, otomicroscopy and middle ear findings were classified as abnormal when fluid was present in the middle ear cavity, the tympanic membrane was retracted, the VT was in place, or the tympanic membrane was perforated. All data were analyzed using the SPSS software (version 22, SPSS Inc., Chicago, IL, USA). Findings were presented as mean±SD for continuous and as frequencies for categorical variables. The Mann-Whitney U test and chi-square test were used to compare continuous and categorical variables. A P-value of less than 0.05 was considered significant.

## Results

Forty children who met the inclusion criteria were included in the study. The basic characteristics of patients are summarized in Table 1. Considering gender, 27 (67.5%) patients were female. In total, 28 (70 %) patients had isolated cleft palate, 9 (22.5%) had unilateral CLP, and 3 (7.5%) had bilateral CLP. Moreover, 17 (42.5%) patients had palatoplasty before their first birthday. The mean age at the time of study and the mean follow-up duration were 7.5 and 5.8 years, respectively. In total, 34 (85%) patients had OME at the time of palatoplasty, 32 (94%) had concurrent VT insertion and palatoplasty, 2 (6%) had sequential VT insertion (VT insertion was before palatoplasty), and 8 (23%) needed more than one VT insertion. Otomicroscopy and tympanometry at the time of study showed 12 patients with abnormal findings: 7 patients had persistent OME, 2 had retracted tympanic membrane, and 3 had perforated tympanic membrane. There was no significant difference in middle ear status between children with complete and incomplete cleft palates (Table 2). The mean age at the time of study and the mean follow-up duration were significantly higher in the normal middle ear group, compared to the abnormal middle ear group (7.7±1.6 vs. 6.8±0.9, P=0.03 and 6±1.15 vs. 5.42±0.9, P=0.04, respectively). Middle ear status was not significantly different between early and late palatoplasty patients. Additionally, the frequency and timing of VT insertion were not significantly different between the two groups. 

**Table 1:** Basic characteristics of patients

**Gender, Female, N (%)**	**27 (67.5%)**
Type of cleft, N (%) Incomplete cleft palate Complete cleft palate	28 (70%) 12 (30%)
Age at the time of the study (range in years)	7.5 (6-12)
Age at palatoplasty Early, <1-year, N (%) (Mean month±SD) Late, ≥1-year, N (%) (Mean month±SD)	17 (42.5%) 10 (1.2) 23 (55.5%) 22 (2.3)
Follow-up duration (mean year±SD)	5.8
Otitis media with effusion at the time of palatoplasty, N (%)	34 (85%)
Timing of ventilation tube insertion Concurrent, N (%) Sequential, N (%)	32 (94%) 2 (6%)
Frequency of VT insertion Once, N (%) ≥2 times, N (%)	26 (76%) 8 (23%)
Normal otoscopy at the time of the study, N (%) Abnormal otoscopy, N (%) Otitis media with effusion (N) Retraction (N) Perforation (N) Cholesteatoma (N) Ventilation tube in place	28 (70%) 12 (30%) 7 2 3 0 0

**Table 2:** Comparison of findings between the two groups

	**Abnormal middle ear** **(N=12)**	**Normal middle ear** **(N=28)**	**P-value**
Gender, Female, N (%)	9 (75%)	18 (64%)	0.23
Type of cleft (N) Incomplete cleft palate Complete cleft palate	7 5	21 7	
Age at the time of the study (mean year±SD)	7.7±1.6	6.8±0.9	0.03
Follow-up duration (mean year±SD)	6±1.15	5.42±0.9	0.04
Age at palatoplasty (N) <1-year (N) ≥1-year (N)	4 8	9 19	0.71
Frequency of ventilation tube insertion (N) once ≥2 times	6 5	20 3	0.07
Timing of ventilation tube insertion (N) Concurrent Sequential	10 1	22 1	0.42

## Discussion

The CLP is a debilitating anomaly for patients and a psychologically stressful condition for parents. The OME, which is commonly associated with CLP, affects the quality of life and educational performance. Numerous factors are involved in the formation of OME in CLP patients. The Eustachian tube of children is shorter, narrower, and more horizontal than that of adults (11,12).

Abnormal forming of the tensor veli palatini muscle and levator veli palatini muscle in children with CLP can cause the malfunctioning of the Eustachian tube. Differences in the mastoid-middle ear-Eustachian tube system in CLP patients are associated with increased recurrent or persistent OME in CLP children (13). 

Currently, there is no general agreement on the treatment of OME. The VT insertion is performed on CLP patients to overcome the middle ear effusion, improve the hearing ability of children, and enhance linguistic development. Several studies reported that in 48.7% to 86% of the CLP patients who underwent VT insertion, OME resolved within the first 6.5 years, and the hearing level of CLP children that underwent early VT insertion was comparable to that of normal children (14-16). However, some researchers recommended watchful waiting for OME in CLP patients. They noted that OME and Eustachian tube function improve as patients become older (17,18). Rynnel-Dagöö et al. described that 82% of CLP patients had a full recovery from OME at 3-4 years of age (19).

The timing of palatoplasty varies among studies, and there are studies discussing the effects of age at palatoplasty on middle ear outcomes. Antonelli et al. explained that age at palatoplasty was not significantly associated with the rate of normal hearing (20). On the other hand, Goh et al. described that patients who had surgery before one year of age had better hearing outcomes (21). 

The hearing outcomes of children with CLP have been associated with the type of cleft palate and the frequency of VT insertion. Some studies showed that CLP children with more than one VT insertion had worse hearing outcomes, compared to those undergoing one VT insertion (9,18). This study evaluated the otologic outcomes in children at least five years post-repair. The findings showed that 30% of CLP children had abnormal middle ear status at the time of the study. The mean age at the time of the study and the mean follow-up duration were significantly higher in patients with normal middle ear status.

Middle ear status was not significantly different between early and late palatoplasty patients. The frequency and timing of VT insertion were not significantly different between the two groups. Moreover, there was no significant difference in middle ear status between children with complete and incomplete cleft palates. Despite considerable research to assess the effectiveness of VT insertion for OME in CLP patients, few studies are using intervelar veloplasty under magnification (Sommerlad’s Technique). It was an advantage that the same technique was used for all patients by the same surgeon. Furthermore, the mean follow-up duration was long. The major limitation of the present study was that obtaining audiology datasets and the hearing threshold of patients was impossible. 

It is recommended that future studies evaluate different factors that influence the hearing outcomes and may affect speech and articulation in CLP children.

## Conclusion

Middle ear status improved as patients grew older but the age of palatoplasty and the frequency of VT insertion were not notable prognostic factors in patients who underwent intervelar veloplasty under magnification.
